# Evaluation and Comparison of the Academic Quality of Open-Access Mega Journals and Authoritative Journals: Disruptive Innovation Evaluation

**DOI:** 10.2196/59598

**Published:** 2025-01-15

**Authors:** Yuyan Jiang, Xue-li Liu, Liyun Wang

**Affiliations:** 1 Henan Research Center for Science Journals Xinxiang Medical University Xinxiang China; 2 Faculty of Humanities & Social Sciences Xinxiang Medical University Xinxiang China

**Keywords:** innovative evaluation, disruption index, open-access mega journals, paper evaluation, open citation data

## Abstract

**Background:**

Some scholars who are skeptical about open-access mega journals (OAMJs) have argued that low-quality papers are often difficult to publish in more prestigious and authoritative journals, and OAMJs may be their main destination.

**Objective:**

This study aims to evaluate the academic quality of OAMJs and highlight their important role in clinical medicine. To achieve this aim, authoritative journals and representative OAMJs in this field were selected as research objects. The differences between the two were compared and analyzed in terms of their level of disruptive innovation. Additionally, this paper explored the countries and research directions for which OAMJs serve as publication channels for disruptive innovations.

**Methods:**

In this study, the journal information, literature data, and open citation relationship data were sourced from Journal Citation Reports (JCR), Web of Science (WoS), InCites, and the OpenCitations Index of PubMed Open PMID-to-PMID citations (POCI). Then, we calculated the disruptive innovation level of the focus paper based on the local POCI database.

**Results:**

The mean Journal Disruption Index (JDI) values for the selected authoritative journals and OAMJs were 0.5866 (SD 0.26933) and 0.0255 (SD 0.01689), respectively, showing a significant difference. Only 1.48% (861/58,181) of the OAMJ papers reached the median level of disruptive innovation of authoritative journal papers (*MD_AJ_*). However, the absolute number was roughly equal to that of authoritative journals. OAMJs surpassed authoritative journals in publishing innovative papers in 24 research directions (eg, Allergy), accounting for 40.68% of all research directions in clinical medicine. Among research topics with at least 10 authoritative papers, OAMJs matched or exceeded *MD_AJ_* in 35.71% of cases. The number of papers published in authoritative journals and the average level of disruptive innovation in each country showed a linear relationship after logarithmic treatment, with a correlation coefficient of –0.891 (*P<*.001). However, the number of papers published in OAMJs in each country and the average level of disruptive innovation did not show a linear relationship after logarithmic treatment.

**Conclusions:**

While the average disruptive innovation level of papers published by OAMJs is significantly lower than that of authoritative journals, OAMJs have become an important publication channel for innovative research in various research directions. They also provide fairer opportunities for the publication of innovative results from limited-income countries. Therefore, the academic community should recognize the contribution and value of OAMJs to advancing scientific research.

## Introduction

Henry Oldenburg, the founding editor of the journal Philosophical Transactions of the Royal Society, noted that the purpose of scholarly journals is to provide a forum for researchers to transfer knowledge to each other and contribute to perfecting the grand design of all the philosophical arts and sciences. However, due to technical limitations, printed journals have been the only form of journal publication since the first journal was introduced in the 17th century. Therefore, researchers must usually wait for 10 to 12 months or more before publishing a paper [1]. The appearance of web-based publications has solved the limitations of page size and publishing cycles. Readers can access the latest research more quickly through online publishing and distribution, which provides a more flexible and efficient way for knowledge dissemination and sharing. This transformation significantly enhanced content deliverability, but accessibility has not kept pace. The urgent need to address this issue has fueled the rise of the open-access (OA) movement and the development of OA journals. Simultaneously, the rapid development of science and technology, combined with the pressure of “publish or perish,” has created an increasing demand for efficient, timely, and cost-effective publication methods. Furthermore, the rapid increase in the number of publications has imposed a growing financial burden on academic institutions and researchers seeking access to scholarly literature. Providing a platform that can accommodate the rapid publication and huge volume of emerging research while facilitating academic exchange and reducing the economic strain of accessing academic resources has become a widespread concern for the scientific community.

Open-access mega journals (OAMJs) may currently be one of the most effective solutions, offering faster publishing speeds, higher acceptance rates [2], and support for the initial exploration of new fields [3]. These advantages align well with the needs of authors [4]. Additionally, many OAMJs provide readers with value-added functions that most traditional journals still lack [5].

Since the establishment of the Public Library of Science (PLoS) in 2001, numerous traditional academic publishers have launched their own OAMJs to expand their product lines [6]. This has positioned OAMJs as increasingly important players in facilitating academic exchanges [7]. However, the evaluation criteria of most OAMJs only pay attention to *research rationality* [8]. Coupled with the financial incentives of OA publishers to publish widely, concerns have arisen about the quality of research published in OAMJs, which have a higher publishing error rate [9] and possibility of predatory publishing [10].

Most OAMJ papers have received a significant number of citations [11], which often exceed the average citation level [12]. However, the evaluation processes of authoritative journals cannot guarantee the accuracy of the research [13]. Despite this, prejudice still exists among some scholars [14], and the complex attitudes within different academic groups have not significantly improved [15].

It is unfair to view OAMJs solely as a negative development. As Professor John Ioannidis of Stanford University [16] points out, several features of OAMJs align with desirable scientific practices. For instance, OAMJs enhance the visibility of academic papers and their broader academic impact. Furthermore, OAMJs reduce selective publication biases by accommodating results that are considered undesirable in traditional professional journals, thus fostering the diversity of perspectives and challenging orthodoxy.

Since 1972, the mainstream evaluation system has relied on citation metrics [17], which means indicators such as the Journal Impact Factor (JIF) have been widely used in academic review, promotion, and tenure evaluation. However, it is important to note that citation metrics primarily reflect the impact of journals rather than their academic innovativeness. Related research has shown that relying on these impact indicators introduces biases against innovation [18], often resulting in flawed decisions in research assessment and evaluation [19].

Disruptive innovation is defined as the process by which a new product, service, or technology transforms an existing market by creating a new market or significantly altering an existing one. This is one of the core driving forces for developing productivity. Therefore, conducting a scientific and reasonable evaluation of the innovation levels in OAMJ papers is crucial for determining their value. Given that clinical medicine is the primary focus of OAMJ papers [5], this paper selects the authoritative journals and the representative OAMJs related to this field as its research subjects. This study compares and analyzes the differences in disruptive innovation levels between these 2 groups and examines the countries and research directions for which OAMJs serve as publication channels for disruptive innovations.

## Methods

### Research Objects

The objects of this study include 2 groups of journals, representing authoritative journals in the field of clinical medicine and OAMJs. The selection criteria for authoritative journals were as follows: (1) JIF ranks high in the field; (2) selected journals are not review journals; and (3) academic authority is widely recognized. The selection criteria for representative OAMJ journals were as follows: (1) OA peer-reviewed journals that charge publication processing fees; (2) an annual average of more than 2000 OA research papers published on clinical medicine topics; (3) a 3-year impact factor within Q1 or Q2.

Through a search of relevant journals in the field of clinical medicine, we discovered that the impact factors of the British Medical Journal (BMJ), the New England Journal of Medicine, The Lancet, and the Journal of the American Medical Association (JAMA) ranked among the highest in impact factors between 2017 and 2019, with widely recognized academic authority [20-25]. We also found that BMJ Open, PLoS ONE, and Scientific Reports published more than 2000 open-access research papers on clinical medicine topics annually, establishing a certain degree of academic influence.

Based on these criteria, this study selected BMJ, New England Journal of Medicine, The Lancet, and JAMA as the authoritative journals and BMJ Open, PLoS ONE, and Scientific Reports as the representative OAMJs.

Because there is no disruptive innovation in review articles, and because Bornmann et al [26] suggest that the disruption index should only be calculated for research papers with at least 10 references, this study exclusively focused on clinical medical research papers with more than 10 references from the aforementioned journals.

### Data Sources

The data required for this study included journal information, literature information, and citation relationship information, obtained through Journal Citation Reports (JCR), Web of Science (WoS), InCites, and the OpenCitations Index of PubMed open PMID-to-PMID citations (POCI), respectively. POCI, a data set sourced from the National Institutes of Health, comprises over 700 million citations and 29 million bibliographic resources. In POCI, citations are regarded as first-class data entities with 7 attribute fields, including the citation time span. To evaluate disruptive innovation, we selected focus papers and used their PMIDs along with POCI data identifiers to calculate the disruptive innovation level for each paper.

### Data Acquisition and Processing

First, we logged into JCR, WoS, and InCites to retrieve the journal information and literature information of the selected journals and save them. Then, we logged into opencitations.net, entered the download page, selected the POCI data set, and downloaded the dump data in CSV format via the Figshare platform. Next, we used Navicat software (PremiumSoft CyberTech Ltd) to import all data into the local SQLite database and process the downloaded POCI data. Finally, in the local database based on the POCI data transformation, we used the PMID numbers extracted from the full records of the focus literature to identify the citation relationship associated with the focus papers. We established relevant data tables for the subsequent calculations.

The disruption index calculation was highly dependent on the time window chosen. The findings of Liu et al [27] confirm that the stabilization time window for the disruption index varies across different disciplines. We believe this is partly due to the different stages of development in various disciplines. Bornmann and Tekles [28] suggest that a time window of 3 years or more is an important prerequisite for ensuring the validity of the calculation results. Given the complexity of the disruption index calculation, the large volume of data [29], and our lack of access to extensive commercial citation data resources, we used the open citation data provided by OpenCitations for the calculation. To guarantee the accuracy of the results of the paper's innovation measurement while minimizing the computational workload, the citation time window in this study was set to 3 years.

### Evaluation Indicators

Since Christensen [30] of Harvard Business School posited the theory of disruptive innovation in 1996, it has become an important paradigm in the field of innovation research. Based on this theory, Wu et al [31] put forward the disruption index (often referred to as the D Index for short), which can be used to measure disruption by calculating the citation substitution of focus papers in the citation network. Based on the disruption index, Bornmann et al [26] discussed the effectiveness of the convergence of this index and the variants that may improve the measurement effect. Ruan et al [32] analyzed the application limitations of the disruption index for measuring progress in the fields of science and technology.

Considering the process of metric calculation, F-type citations reflect that the focus paper disrupts the reference, B-type citations indicate that the focus paper is a development of the reference, and R-type citations reveal that the focus paper inherits the reference. Liu et al [33] argued that the disruption Index is a “relative” concept because it measures disruption based on the relative size of *N_F_* (F-type citations) and *N_B_* (B-type citations). They defined the disruption index, which is only reflected by F-type citations, as the “absolute disruption index.” Additionally, Liu et al [27,34] empirically studied the stabilization time window for the disruption index in different subject areas and resolved mathematical inconsistencies in its traditional formation (eg, *N_R_* not satisfying monotonicity).

Based on this disruption index, Bornmann et al [35], Horen et al [36], Sullivan et al [37], Meyer et al [38], Jiang and Liu [39], and others have mined disruptive papers in the fields of scientometrics, craniofacial surgery, pediatric surgery, synthetic biology, and energy security respectively. On this basis, Jiang and Liu [40] proposed a method for calculating the Disruption index using open citation data during previous research. They carried out evaluations at both the literature and journal levels [41-45], contributing to a mature framework for evaluating disruptive innovation.



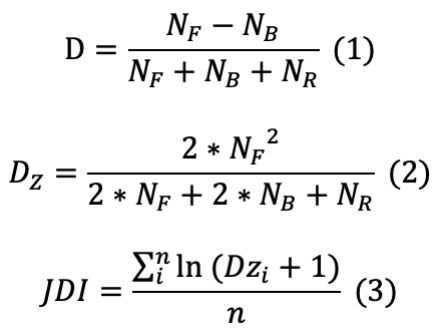



In equations (1) to (3), *N_F_* refers to the literature that only cites the focus paper (FP); *N_B_* refers to the literature that cites both the focus paper and at least one reference (R) of the focus paper; *N_R_* refers to literature that only cites at least one reference (R) of the focus paper but not the focus paper; n is the number of articles contained in the journal; and *D_Zi_* is the absolute disruption index (*D_Z_*) of the i^th^ article in the journal.

## Results

### Comparative Analysis of the Journals’ Disruptive Innovation Levels

Annual changes in JIF, JDI, and the number of published articles for the 7 journals selected in this study between 2017 and 2019 are shown in Table 1. The key findings are as follows. First, the average JDI of the selected authoritative journals is 0.5866, while the average JDI of OAMJs is 0.0255, indicating a significant difference. Second, the JDI values of the authoritative journals are higher than those of OAMJs. Third, JDI does not necessarily increase with a higher JIF.

Additionally, to better measure the difference in disruptive innovation level between OAMJ papers and authoritative journal papers, this study defined 2 parameters: the median *D_Z_* of authoritative journals (*MD*_AJ_) and the median *D_Z_* of OAMJs (*MD*_OAMJ_), which were 0.289 and 0.0019, respectively. The findings indicated that (1) the disruptive innovation level (94.60 %) of authoritative journal papers was higher than *MD*_OAMJ_ and (2) only 1.48% (861/58151) of OAMJ papers had a disruptive innovation level higher than *MD*_AJ_. However, the number of OAMJ papers (n=861, 1.48%) was close to half of the number of authoritative journals (n=1128 of 2222).

**Table 1:** Annual changes in JIF^a^, JDI^b^, and number of articles for the 7 selected journals.

Journal	JIF				JDI				Number of articles					Number of COCI^c^ articles			
	2017	2018	2019	2017		2018	2019	2017		2018	2019	All	2017		2018	2019	All
BMJ^d^	23.259	27.604	30.223	0.2691		0.1897	0.2580	140		147	140	427	123		126	124	373
NEJM^e^	31.398	36.216	38.637	0.5276		0.3811	0.4584	275		276	277	828	258		270	265	793
The Lancet	53.254	59.102	60.392	0.7459		0.7132	0.6682	242		191	192	625	233		183	187	603
JAMA^f^	79.258	70.67	74.699	0.9268		1.0325	0.8684	179		147	157	483	177		136	140	453
BMJ Open	2.413	2.376	2.496	0.0730		0.0211	0.0210	2160		2112	3512	7784	2055		2025	3345	7425
PLoS^g^ ONE	2.766	2.776	2.740	0.0180		0.0211	0.0190	19,976		17,461	14,880	52,317	10,444		9153	7892	27,489
Scientific Reports	4.122	4.011	3.988	0.0161		0.0223	0.0180	24,806		17,118	19,843	61,767	9071		6543	7653	23,267

^a^JIF: Journal Impact Factor.

^b^JDI: Journal Disruption Index.

^c^COCI: OpenCitations Index of CrossRef Open DOI-to-DOI Citations

^d^BMJ: British Medical Journal.

^e^NEJM: New England Journal of Medicine.

^f^JAMA: Journal of the American Medical Association.

^g^PLoS: Public Library of Science.

### Comparative Analysis of Disruptive Innovation Level Across Fields of Paper

Due to the complexity and diversity of clinical medical research, this study summarized statistics based on the classification results of InCites to assess the disruption of OAMJ papers and authoritative journal papers across various research fields. The findings, shown in Table 2, revealed several key insights.

There were differences in the average levels of disruptive innovation among OAMJ papers published in different research fields. The highest level was observed in the research field of immunology (0.1965), while neuroimaging had the lowest level (0.0049).

Similarly, there were differences in the average levels of disruptive innovation in authoritative journal papers, with reproductive biology showing the highest level (15.7902) and sport sciences (0.0031) showing the lowest level.

Across all fields, authoritative journal papers outperformed OAMJ papers in terms of disruptive innovation at the overall level. In 24 research directions such as allergy—accounting for 40.68% of all clinical medicine research areas—the number of OAMJ papers with disruptive innovation levels exceeding *MD*_AJ_ surpassed half the number of authoritative journal papers. Among the topics with no less than 10 authoritative papers published, the topics in which the number of papers published by OAMJ with a level of disruptive innovation exceeding *MD*_AJ_'s was no less than half of the number of papers published in authoritative journals accounted for 35.71% of the total.

**Table 2:** Comparative analysis of disruptive innovation level across fields of paper.

Field	Authoritative journals					OAMJ^a^			
	Amount	Average (*D*_*z*_^b^)	MID^c^ (*D*_*z*_)	Amount (*Dz*>*MD*_*OAMJ*_^d^), n (%)	Amount		Average (*D*_z_)	MID (*D*_*z*_)	Amount (*D*_*z*_>*MD*_AJ_^*e*^), n (%)
Allergy	4	0.2268	0.0481	3 (75)	32		0.0264	0.0028	2 (6.25)
Andrology	0	—^f^	—	—	2		0.0408	0.0027	—
Anesthesiology	16	0.8493	0.4280	16 (100)	280		0.0247	0.0015	2 (0.71)
Audiology and speech-language pathology	0	—	—	—	63		0.0136	0	—
Behavioral sciences	0	—	—	—	256		0.0218	0.0017	—
Cardiac and cardiovascular systems	217	3.1848	0.2502	211 (97.24)	2510		0.0141	0.0015	15 (0.60)
Clinical neurology	146	3.2995	0.3190	138 (94.52)	1834		0.0149	0.0012	10 (0.55)
Critical care medicine	57	1.6419	0.2253	57 (100)	563		0.0164	0.0012	10 (1.78)
Dentistry, oral surgery and medicine	2	2.1744	1.2166	2 (100)	607		0.0288	0.0039	0 (0)
Dermatology	18	2.4297	0.4658	18 (100)	299		0.0400	0.0038	4 (1.34)
Emergency medicine	8	1.3246	0.1213	8 (100)	196		0.0508	0.0024	12 (6.12)
Endocrinology and metabolism	134	2.8454	0.2190	128 (95.52)	2583		0.0215	0.0024	45 (1.74)
Engineering, biomedical	0	­—	—	—	467		0.0418	0.0052	—
Gastroenterology and hepatology	65	2.2936	0.3151	62 (95.38)	1769		0.0245	0.0025	20 (1.13)
Genetics and heredity	31	0.8752	0.1720	28 (90.32)	2247		0.0062	0.0002	11 (0.49)
Geriatrics and gerontology	10	1.1717	0.3157	10 (100)	321		0.0327	0.0018	5 (1.56)
Health care sciences and services	21	4.0306	0.2428	21 (100)	732		0.0538	0.0027	29 (3.96)
Health policy and services	5	4.3698	2.9845	4 (80)	92		0.0646	0.0054	0 (0)
Hematology	72	2.9323	0.7213	65 (90.28)	689		0.0158	0.0015	2 (0.29)
Immunology	44	2.6776	0.3863	40 (90.91)	3432		0.1965	0.0022	18 (0.52)
Infectious diseases	78	7.1637	0.3698	70 (89.74)	1969		0.0460	0.0043	49 (2.49)
Integrative and complementary medicine	0	—	—	—	25		0.0996	0.0043	—
Materials science, biomaterials	0	—	—	—	183		0.0716	0.0152	—
Medical ethics	0	—	—	—	1		0.0131	0.0131	—
Medical informatics	1	0.2245	0.2245	1 (100)	30		0.1121	0.0069	3 (10)
Medical laboratory technology	1	0.2120	0.2120	1 (100)	25		0.0115	0.0013	0 (0)
Medicine, general and internal	357	6.2389	0.3111	335 (93.84)	1415		0.1411	0.0028	68 (4.81)
Medicine, legal	0	—	—	—	57		0.0163	0.0013	—
Medicine, research and experimental	4	8.5306	0.1741	4 (100)	63		0.0638	0.0067	3 (4.76)
Neuroimaging	0	—	—	—	3		0.0049	0.0025	—
Neurosciences	9	1.0562	0.4969	9 (100)	7794		0.0094	0.0008	9 (0.12)
Nursing	0	—	—	—	159		0.0638	0.0047	—
Nutrition and dietetics	29	1.0717	0.1605	28 (96.55)	764		0.0300	0.0031	28 (3.66)
Obstetrics and gynecology	89	1.6846	0.1264	79 (88.76)	1019		0.0349	0.0028	54 (5.30)
Oncology	207	8.9447	1.3285	199 (96.14)	6032		0.0337	0.0025	13 (0.22)
Ophthalmology	19	4.7031	0.1073	18 (94.74)	1776		0.0248	0.0019	71 (4)
Orthopedics	22	0.7836	0.0998	22 (100)	658		0.0206	0.0021	21 (3.19)
Otorhinolaryngology	6	0.9314	0.0747	6 (100)	244		0.0249	0.0027	15 (6.15)
Pathology	2	0.0575	0	1 (50)	57		0.0139	0.0045	57 (100)
Pediatrics	66	0.6416	0.1569	63 (95.45)	667		0.0316	0.0020	26 (3.90)
Peripheral vascular disease	25	1.2093	0.1844	23 (92)	616		0.0175	0.0025	9 (1.46)
Pharmacology and pharmacy	4	1.5833	1.4107	4 (100)	1141		0.0612	0.0065	6 (0.53)
Primary health care	0	—	—	—	60		0.1677	0.0099	—
Psychiatry	50	0.2403	0.0509	43 (86)	1511		0.0378	0.0013	96 (6.35)
Psychology, clinical	3	0.4601	0.3300	3 (100)	162		0.0075	0.0005	0 (0)
Public, environmental, and occupational health	139	3.8125	0.3887	132 (94.96)	3861		0.0507	0.0044	92 (2.38)
Radiology, nuclear medicine, and medical imaging	8	1.4056	0.2714	7 (87.50)	1418		0.0303	0.0021	22 (1.55)
Rehabilitation	1	0.3139	0.3139	1 (100)	256		0.0340	0.0010	4 (1.56)
Reproductive biology	1	15.7902	15.7902	1 (100)	363		0.0159	0.0022	0 (0)
Respiratory system	76	1.4090	0.2322	73 (96.05)	934		0.0248	0.0019	14 (1.50)
Rheumatology	50	1.8720	0.1174	44 (88)	738		0.0191	0.0021	18 (2.44)
Sport sciences	2	0.0031	0	1 (50)	780		0.0290	0.0016	780 (100)
Substance abuse	11	9.7414	0.1482	10 (90.91)	191		0.0268	0.0040	7 (3.66)
Surgery	47	1.0035	0.3107	45 (95.74)	797		0.0310	0.0025	15 (1.88)
Toxicology	1	0.0081	0.0081	1 (100)	312		0.0308	0.0029	111 (35.58)
Transplantation	5	0.0464	0.0303	4 (80)	82		0.0134	0.0019	9 (10.98)
Tropical medicine	7	1.4192	0.6969	6 (85.71)	268		0.0311	0.0039	1 (0.37)
Urology and nephrology	56	2.7370	0.1476	52 (92.86)	1573		0.0175	0.0019	37 (2.35)
Virology	2	6.0473	1.6838	2 (100)	1212		0.0107	0.0006	0 (0)

^a^OAMJ: open-access mega journal.

^b^*D_Z_*: absolute disruption index.

^c^MID: median.

^d^*MD_OAM_*_J_: median *D_Z_* of the OAMJ papers.

^e^*MD*_AJ_: median *D_Z_* of the authoritative journal papers.

^f^Not applicable.

### Comparative Analysis of Disruptive Innovation Level of Papers Across Countries

The disruptive innovation levels of OAMJ papers and authoritative journal papers published by different countries are shown in Tables 3 and 4, which include data for countries with records in both types of journals. To better describe the relevant results, this study used the following criteria. First, the number of authoritative journal papers is used as a measure of national scientific research strength. A higher number of published authoritative papers reflects stronger national scientific research capabilities. Second, the difference in the disruptive innovation levels between published OAMJ papers and authoritative journal papers serves as a measure of the recognition of OAMJs by scholars in different countries. A smaller difference indicates a higher degree of recognition. Third, the ratio of OAMJ papers to authoritative journal papers measures the degree of favoritism scholars from different countries show toward OAMJs. A larger ratio suggests a higher degree of favoritism toward OAMJs by scholars from a particular country.

From Tables 3 and 4, we observed several key insights. First, there was a difference between the average disruptive innovation level of OAMJ papers across countries, with South Korea ranking the highest (0.5476) and Serbia ranking the lowest (0.0043). Similarly, the average disruptive innovation level of authoritative journal papers also varied across countries, with Peru ranking the highest (35.6125) and Serbia ranking the lowest (4.7261).

**Table 3:** Comparison of papers published in different countries.

Country	Avg^a^ (*DZ* ^b^_*AJ*_^c^)	Amount of AJ articles, n (%)	Amount ≥ *MD*_OAMJ_^d^, n (%)	Avg *(DZ*_*OAMJ*_*)*	Amount of OAMJ articles, n (%)	Amount *D*_*z*_≥*MD*_AJ_^e^, n (%)	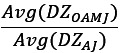	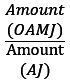
United States	4.7261	1537 (76.01)	1435 (93.36)	0.0588	1499 (2.58)	60 (4)	0.0124	0.9753
United Kingdom	5.4159	833 (41.20)	818 (98.20)	0.1113	2967 (5.10)	178 (6)	0.0205	3.5618
Canada	7.5419	479 (23.69)	471 (98.33)	0.0607	845 (1.45)	27 (3.20)	0.0080	1.7641
Germany	8.5920	399 (19.73)	396 (99.25)	0.0538	517 (0.89)	12 (2.32)	0.0063	1.2957
Australia	9.7657	355 (17.56)	350 (98.59)	0.0797	1336 (2.30)	33 (2.47)	0.0082	3.7634
France	11.2194	340 (16.82)	338 (99.41)	0.0757	371 (0.64)	13 (3.50)	0.0068	1.0912
Netherlands	8.2619	296 (14.64)	291 (98.31)	0.0563	648 (1.11)	19 (2.93)	0.0068	2.1892
Italy	9.9492	275 (13.60)	274 (99.64)	0.1065	255 (0.44)	12 (4.71)	0.0107	0.9273
Spain	12.6857	227 (11.23)	225 (99.12)	0.0380	297 (0.51)	6 (2.02)	0.0030	1.3084
Switzerland	10.9062	216 (10.68)	215 (99.54)	0.1126	265 (0.46)	13 (4.91)	0.0103	1.2269
Sweden	8.9276	191 (9.45)	185 (96.86)	0.0346	490 (0.84)	13 (2.65)	0.0039	2.5654
Denmark	8.9724	169 (8.36)	165 (97.63)	0.0761	381 (0.65)	12 (3.15)	0.0085	2.2544
China Mainland	13.1854	167 (8.26)	166 (99.40)	0.0738	945 (1.62)	47 (4.97)	0.0056	5.6587
Japan	17.6698	166 (8.21)	161 (96.99)	0.0439	319 (0.55)	7 (2.19)	0.0025	1.9217
Belgium	13.5063	160 (7.91)	158 (98.75)	0.1137	202 (0.35)	9 (4.46)	0.0084	1.2625
Brazil	14.4105	139 (6.87)	135 (97.12)	0.0448	147 (0.25)	7 (4.76)	0.0031	1.0576
Poland	12.6655	128 (6.33)	128 (100)	0.0254	80 (0.14)	2 (2.50)	0.0020	0.6250
South Korea	17.2768	117 (5.79)	117 (100)	0.5476	181 (0.31)	4 (2.21)	0.0317	1.5470
New Zealand	12.9764	103 (5.09)	100 (97.09)	0.1546	231 (0.40)	4 (1.73)	0.0119	2.2427
South Africa	15.5478	98 (4.85)	97 (98.98)	0.0410	163 (0.28)	6 (3.68)	0.0026	1.6633
India	15.8468	95 (4.70)	94 (98.95)	0.0913	154 (0.26)	10 (6.49)	0.0058	1.6211
Norway	15.7190	92 (4.55)	88 (95.65)	0.0238	334 (0.57)	8 (2.40)	0.0015	3.6304
Israel	11.6714	87 (4.30)	86 (98.85)	0.0189	43 (0.07)	1 (2.33)	0.0016	0.4943
Finland	13.9462	84 (4.15)	83 (98.81)	0.1585	161 (0.28)	7 (4.35)	0.0114	1.9167
Austria	16.6162	83 (4.10)	83 (100)	0.2537	65 (0.11)	2 (3.08)	0.0153	0.7831
Russia	17.6035	77 (3.81)	77 (100)	0.0259	12 (0.02)	0 (0)	0.0015	0.1558
Ireland	12.5726	76 (3.76)	76 (100)	0.0607	189 (0.32)	8 (4.23)	0.0048	2.4868
Taiwan	19.8153	68 (3.36)	68 (100)	0.0245	213 (0.37)	4 (1.88)	0.0012	3.1324
Czech Republic	10.1522	65 (3.21)	65 (100)	0.0464	21 (0.04)	1 (4.76)	0.0046	0.3231
Singapore	17.4077	65 (3.21)	65 (100)	0.0808	106 (0.18)	4 (3.77)	0.0046	1.6308
Argentina	15.2021	61 (3.02)	60 (98.36)	0.1178	18 (0.03)	3 (16.67)	0.0077	0.2951
Turkey	19.7073	61 (3.02)	61 (100)	0.0983	21 (0.04)	4 (19.05)	0.0050	0.3443
Hong Kong	18.6193	58 (2.87)	57 (98.28)	0.0208	93 (0.16)	2 (2.15)	0.0011	1.6034
Mexico	25.8280	58 (2.87)	57 (98.28)	0.0132	33 (0.06)	0 (0)	0.0005	0.5690
Chile	19.7926	52 (2.57)	52 (100)	0.1587	37 (0.06)	6 (16.22)	0.0080	0.7115
Colombia	25.5139	52 (2.57)	52 (100)	0.0752	17 (0.03)	2 (11.76)	0.0029	0.3269
Greece	16.5438	52 (2.57)	52 (100)	0.0681	40 (0.07)	2 (5)	0.0041	0.7692
Portugal	26.4890	52 (2.57)	52 (100)	0.2982	57 (0.10)	4 (7.02)	0.0113	1.0962
Hungary	15.0707	51 (2.52)	51 (100)	0.0099	20 (0.03)	0 (0)	0.0007	0.3922
Malaysia	18.8086	47 (2.32)	47 (100)	0.0523	73 (0.13)	4 (5.48)	0.0028	1.5532
Kenya	22.9862	43 (2.13)	40 (93.02)	0.0629	60 (0.10)	3 (5)	0.0027	1.3953
Pakistan	25.5705	41 (2.03)	40 (97.56)	0.1181	47 (0.08)	7 (14.89)	0.0046	1.1463
Ukraine	27.0706	37 (1.83)	37 (100)	0.0079	4 (0.01)	0 (0)	0.0003	0.1081
Iran	29.1602	36 (1.78)	36 (100)	0.0611	49 (0.08)	3 (6.12)	0.0021	1.3611
Romania	31.1367	36 (1.78)	36 (100)	0.0210	13 (0.02)	0 (0)	0.0007	0.3611
Peru	35.6125	35 (1.73)	35 (100)	0.0118	11 (0.02)	0 (0)	0.0003	0.3143
Nigeria	33.4455	34 (1.68)	34 (100)	0.0410	24 (0.04)	2 (8.33)	0.0012	0.7059
The Philippines	25.4935	32 (1.58)	32 (100)	0.2619	17 (0.03)	3 (17.65)	0.0103	0.5313
Bangladesh	30.1269	31 (1.53)	31 (100)	0.0815	78 (0.13)	9 (11.54)	0.0027	2.5161
Serbia	29.4950	31 (1.53)	31 (100)	0.0043	6 (0.01)	0 (0)	0.0001	0.1935

^a^Avg: average.

^b^*D_Z_*: absolute disruption index.

^c^AJ*:* authoritative journal.

^d^*MD*_OAMJ_: median *D_Z_* of the open-access mega journal (OAMJ) papers.

^e^*MD*_AJ_: median *D_Z_* of the authoritative journal papers.

**Table 4:** Indicator correlation analysis.

Category			AJ^a^									OAMJ^b^									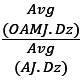			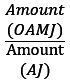
			Avg^c^ (*D_z_*^d^)		Amount		Amount (*D_z_*>MD_OAMJ_^e^)		Proportion (*D_z_*>MD_OAMJ_)		Avg (*D_z_*)			Amount		Amount (*D_z_*>*MD*_AJ_^f^)		Proportion (*D_z_*>*MD*_AJ_)						
**AJ**																								
	Avg (*D*_*z*_)	1		–0.1494		–0.1452		0.359**		–0.1118			–0.265**		–0.256**		–0.0809		–0.461**			–0.322**		
	Amount			1		1**		–0.514**		0.518**			0.895**		0.744**		0.459**		0.513**			0.448**		
	Amount (*D*_*z*_>*MD*_OAMJ_)					1		–0.503**		0.518**			0.894**		0.743**		0.459**		0.512**			0.444**		
	Proportion (*D*_*z*_>*MD*_OAMJ_)							1		–0.196*			–0.537**		–0.497**		–0.197*		–0.338**			–0.448**		
**OAMJ**																								
	Avg (*D*_*z*_)									1			0.579**		0.732**		0.827**		0.878**			0.463**		
	Amount												1		0.806**		0.523**		0.615**			0.764**		
	Amount (*D*_*z*_>*MD*_AJ_)														1		0.821**		0.711**			0.616**		
	Proportion (*D*_*z*_>*MD*_AJ_)																1		0.689**			0.398**		
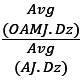																				1			0.553**	
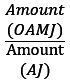																							1	

^a^AJ: authoritative journal.

^b^OAMJ: open-access mega journal.

^c^Avg: average.

^d^*D_Z_*: absolute disruption index.

^e^*MD*_OAMJ_: median *D_Z_* of the OAMJ papers.

^f^*MD*_AJ_: median *D_Z_* of the authoritative journal papers.

* *P*<.01

**P<.001

**Figure 1:**
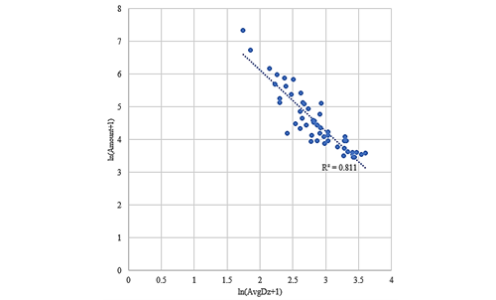
Distribution of the number of publications in authority journals with average disruptive innovation levels among different countries.

**Figure 2:**
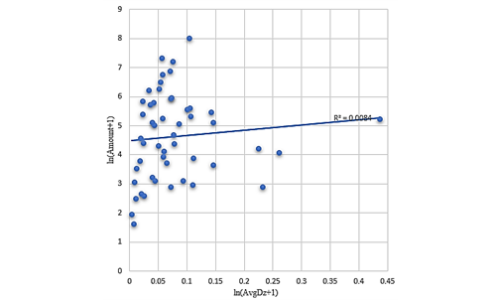
Distribution of the number of publications in open-access mega journals (OAMJs) with the average disruptive innovation levels among different countries.

There was a linear relationship between the number of authoritative journal articles and the average disruptive innovation level of each country after logarithmization (as shown in Figure 1), with a correlation coefficient of –0.891, which indicated that authoritative journals did not consistently recognize the work of scholars from different countries. However, for OAMJ papers issued by each country, the average level of disruptive innovation did not show a linear relationship after logarithmic processing (as shown in Figure 2).

Scholars from different countries also had varying levels of recognition for OAMJs and tendencies to publish them. A higher recognition of OAMJs correlated with a higher likelihood of publishing papers in these journals (0.553). This was significantly correlated with the disruptive innovation levels of both authoritative journal papers (–0.461) and OAMJ papers (0.878). Moreover, the proportion of OAMJ papers meeting the *MD*_AJ_ threshold was significantly and positively correlated with the tendency to publish papers in OAMJs (0.689).

Among all countries, those that published more than 10% of OAMJ papers with a level of disruptive innovation above *MD*_AJ_, while maintaining an average annual publication count of at least 1 in the selected journals, included Argentina, Turkey, Chile, Colombia, Pakistan, Philippines, Bangladesh, and Egypt.

## Discussion

### Principal Results

#### Differences Between the Disruptive Innovation Levels of OAMJ and Authoritative Journals

In this study, the disruptive innovation levels of OAMJs were lower than those of authoritative journals. A plausible explanation is that OAMJs require more rationality than innovation during the review process. Therefore, OAMJ papers were more focused on incremental innovation and proximity innovation, while authoritative journals were more likely to publish papers with breakthrough innovation.

#### OAMJs As Important Publishing Platforms for Innovative Research Across Fields

In this study, the overall disruptive innovation levels of authoritative journal papers were higher than those of OAMJ papers across all clinical medical research fields. However, the number of OAMJ papers with a higher level of disruptive innovation than *MD*_AJ_ was close to half of the number of authoritative journal papers. Moreover, in almost half of the research directions examined, the number of OAMJ papers with a higher level of disruptive innovation than *MD*_AJ_ exceeded half of the count for authoritative journal papers.

This phenomenon indicates that the contribution of OAMJs to the overall development of science rivals that of authoritative journals. While OAMJs must continue to emphasize overall quality control, their contribution to scientific development should not be overlooked [46].

Additionally, OAMJ papers involve some research directions that are not covered by authoritative journal papers. Therefore, it is unreasonable to judge the level of disruptive innovation of a paper based solely on the source journal without distinguishing the research direction. Thus, we must evaluate the contributions of OAMJs fairly and scientifically [47].

#### OAMJs Provide a Fair Publishing Channel for Innovative Achievements from Limited-Income Countries

Research has confirmed that national bias exists in the peer-review process [48], and specific journals have preferences for their authors’ countries of origin [49]. Therefore, authoritative journals demonstrate varying degrees of recognition for the research results of scholars from different countries. This phenomenon was also evident in this study, where notable differences were observed in the disruptive innovation levels of authoritative journal papers published by scholars from different countries. Furthermore, the disruptive innovation levels in authoritative journals from each country were linearly and negatively correlated with the volume of publications.

A reasonable explanation for this is the first-mover advantage of high-income countries in the field of science and technology. These nations benefit from a group of senior scholars in various fields whose work often receives recognition that exceeds its quality in the current peer-review process [50]. This recognition also extends to the young scholars they collaborate with [51]. On the other hand, this study also found that OAMJs play an important role in promoting innovation diffusion in limited-income countries. Therefore, OAMJs may provide fairer publishing opportunities, and in tandem with postpublication peer review [52], may contribute to the accelerated development of science and technology.

### Limitations

This study has some limitations that must be acknowledged. First, participating authors with different ranks were treated as equal contributors, disregarding variations. Second, all focus papers were categorized under a single subject classification, without considering their inherent interdisciplinary properties. Third, only the knowledge diffusion of focus papers within the scope of periodical literature was considered, ignoring other venues. These limitations must be explored in future research based on natural language processing technology, and more accurate results can be obtained by jointly using multiple types of citation data sources.

### Conclusions

The disruptive innovation levels of OAMJs were different from those of authoritative journals. However, OAMJs have become an important publication channel for innovative results in several research directions, providing fairer opportunities for researchers from limited-income countries to publish their work. Therefore, the academic community should acknowledge and appreciate the contributions of OAMJs to scientific research.

## Abbreviations

BMJ: British Medical Journal
DZ: absolute disruption index
JAMA: Journal of the American Medical Association
JCR: Journal Citation Reports
JDI: Journal Disruption Index
JIF: Journal Impact Factor
MDAJ: the median of authoritative journal papers
MDOAMJ: the median of open-access mega journal papers
OA: open access
OAMJ: open-access mega journal
PLoS: Public Library of Science
POCI: OpenCitations Index of PubMed open PMID-to-PMID citations
WoS: Web of Science
